# Effect of inoculum density on human‐induced pluripotent stem cell expansion in 3D bioreactors

**DOI:** 10.1111/cpr.12604

**Published:** 2019-05-08

**Authors:** Selina Greuel, Güngör Hanci, Mike Böhme, Toshio Miki, Frank Schubert, Michael Sittinger, Carl‐Fredrik Mandenius, Katrin Zeilinger, Nora Freyer

**Affiliations:** ^1^ Bioreactor Group, Berlin‐Brandenburg Center for Regenerative Therapies (BCRT) Charité – Universitätsmedizin Berlin Berlin Germany; ^2^ Department of Surgery, Keck School of Medicine University of Southern California Los Angeles California; ^3^ StemCell Systems GmbH Berlin Germany; ^4^ Tissue Engineering Laboratory, Berlin‐Brandenburg Center for Regenerative Therapies (BCRT), Department of Rheumatology and Clinical Immunology Charité – Universitätsmedizin Berlin Berlin Germany; ^5^ Division of Biotechnology, Department of Physics, Chemistry and Biology (IFM) Linköping University Linköping Sweden

**Keywords:** 3D culture, bioreactor culture, cell expansion, human‐induced pluripotent stem cells, inoculum density

## Abstract

**Objective:**

For optimized expansion of human‐induced pluripotent stem cells (hiPSCs) with regards to clinical applications, we investigated the influence of the inoculum density on the expansion procedure in 3D hollow‐fibre bioreactors.

**Materials and Methods:**

Analytical‐scale bioreactors with a cell compartment volume of 3 mL or a large‐scale bioreactor with a cell compartment volume of 17 mL were used and inoculated with either 10 × 10^6^ or 50 × 10^6^ hiPSCs. Cells were cultured in bioreactors over 15 days; daily measurements of biochemical parameters were performed. At the end of the experiment, the CellTiter‐Blue^®^ Assay was used for culture activity evaluation and cell quantification. Also, cell compartment sections were removed for gene expression and immunohistochemistry analysis.

**Results:**

The results revealed significantly higher values for cell metabolism, cell activity and cell yields when using the higher inoculation number, but also a more distinct differentiation. As large inoculation numbers require cost and time‐extensive pre‐expansion, low inoculation numbers may be used preferably for long‐term expansion of hiPSCs. Expansion of hiPSCs in the large‐scale bioreactor led to a successful production of 5.4 × 10^9^ hiPSCs, thereby achieving sufficient cell amounts for clinical applications.

**Conclusions:**

In conclusion, the results show a significant effect of the inoculum density on cell expansion, differentiation and production of hiPSCs, emphasizing the importance of the inoculum density for downstream applications of hiPSCs. Furthermore, the bioreactor technology was successfully applied for controlled and scalable production of hiPSCs for clinical use.

## 1 INTRODUCTION

The application of human‐induced pluripotent stem cells (hiPSCs) has shown high potential in the field of clinical therapies^1^ and pharmaceutical drug development,^2^ as this cell type is suitable for generating disease‐specific models and patient‐specific therapies.^3^, ^4^, ^5^, ^6^ However, the utilization of hiPSC models in drug discovery requires high cell quantities of hiPSCs and their derivatives at a constant quality.^7^, ^8^ This can hardly be achieved by using conventional 2D cell cultures due to insufficient cell production yields, lack in scalability and difficulty of controlling cell culture parameters.^9^, ^10^ In contrast, the use of 3D culture models offers the opportunity of large‐scale expansion of hiPSCs under controlled conditions.^9^, ^11^ For production of large cell quantities fulfilling the required quality standards, it is important to consider those factors that potentially influence hiPSC expansion and differentiation in 3D culture systems. Such factors include feeding strategies, coating materials, culture media and the cell inoculum density.^9^ In the present study, the effect of the inoculum density on cell expansion and differentiation of hiPSCs cultured in perfused hollow‐fibre‐based 3D bioreactors was investigated. For this purpose, 10 × 10^6^ hiPSCs resp. 3.3 × 10^6^ cells/mL, or 50 × 10^6^ hiPSCs resp. 16.6 × 10^6^ cells/mL were inoculated into analytical‐scale bioreactors with a cell compartment volume of 3 mL (AS) and cultured over a period of 15 days. Both conditions were compared in terms of biochemical parameters, cell activity and cell yields, gene expression analysis and immunohistochemical staining. Changes in the differentiation state of hiPSCs expanded in bioreactors were detected by gene expression and immunofluorescence analysis, where hiPSCs forming embryoid bodies served as differentiation control. The feasibility of scaling up of hiPSC expansion was tested in a large‐scale 3D bioreactor with a cell compartment volume of 17 mL (LS) using an inoculation number of 50 × 10^6^ cells, resp. 2.9 × 10^6^ cells/mL.

## 2 MATERIALS AND METHODS

### 2.1 Bioreactor system/technology

**Figure 1:**
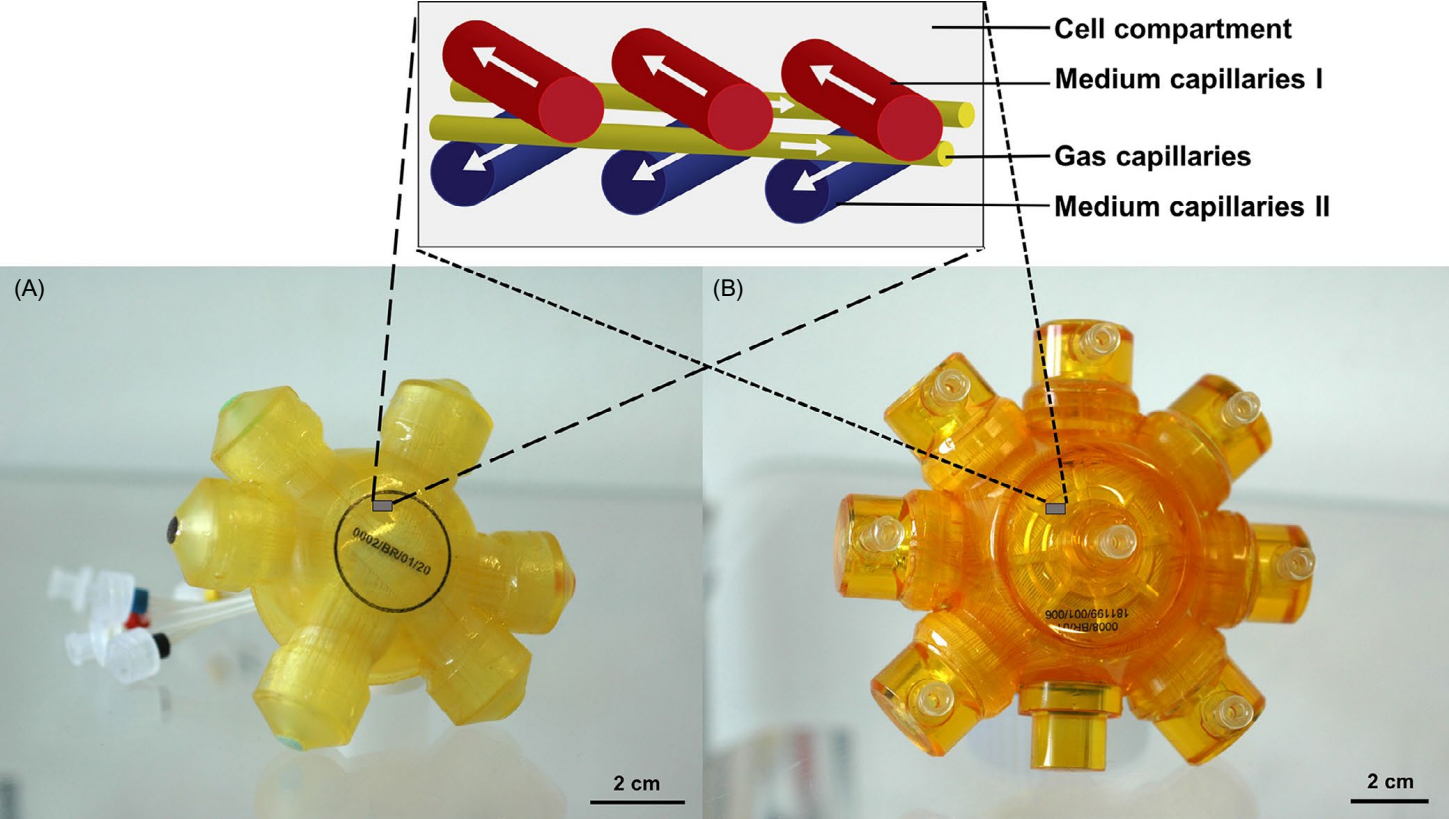
Bioreactor types used for the expansion of hiPSCs with their capillary structure. The picture shows the analytical‐scale bioreactor (A) with a cell compartment volume of 3 mL and the large‐scale bioreactor (B) with a cell compartment volume of 17 mL. The schematic image on top shows a section of the capillary structure inside the bioreactor, consisting of the following four compartments: medium capillaries I (red) and II (blue) for countercurrent medium perfusion, gas capillaries (yellow) and the space surrounding the capillaries, which serves as the cell compartment (white). The scale bars correspond to 2 cm

The 3D four‐compartment hollow‐fibre bioreactor used in this study is based on three independent, interwoven hollow‐fibre capillary bundles, two for supplying nutrient media by countercurrent perfusion and one for gas exchange. The space between these capillary bundles (extracapillary space) serves as cell compartment. The capillary system is integrated into a polyurethane housing. The cells, grown in the cell compartment, were constantly supplied with nutrients and oxygen. The bioreactor types used in this study had a cell compartment volume of 3 mL (analytical‐scale, AS) or 17 mL (large‐scale, LS); specific data regarding compartment measurements as well as perfusion conditions are displayed in Table  1. Both bioreactor types, the AS and LS bioreactor, are constructed identically in respect of their capillary configuration (Figure 1); they only differ in length and number of capillaries. A detailed description of the bioreactor technology can be found elsewhere.^12^, ^13^ The bioreactors were connected to a perfusion device consisting of pumps for medium feed and medium recirculation.

**Table 1:** Specifications of bioreactor compartments and perfusion parameters

	Analytical‐scale	Large‐scale
Volume of bioreactor compartments		
Total inner volume of the bioreactor	5.1 cm^3^	26 cm^3^
Total volume of capillaries	2.2 cm^3^	8.9 cm^3^
Volume of cell compartment	2.9 cm^3^	17.1 cm^3^
Perfusion parameters		
Recirculation rate	10 mL/min	20 mL/min
Feed rate	1‐12 mL/h	2‐40 mL/h
Air	20 mL/min	40 mL/min
CO_2_	0‐1 mL/min	0‐2 mL/min

### 2.2 Pre‐expansion of hiPSCs in 2D cultures

The hiPSC line DF6‐9‐9T^14^ (WiCell Research Institute, Madison, WI, USA) was cultured feeder‐free on six‐well culture plates or T175 culture flasks (both BD Falcon, San José, CA, USA), which were pre‐coated with 8.68 µg/cm^2 ^Matrigel (growth factor reduced, Corning, NY, USA). The culture medium mTeSR^TM^1 (Stemcell Technologies, Vancouver, BC, Canada) was used, supplemented with 10 000 units/mL penicillin and 10 mg/mL streptomycin (Pen Strep, Gibco^®^ by Life Technologies/Thermo Fisher Scientific). After thawing, 1 mmol/L ROCK inhibitor (Y‐27632; Abcam, Cambridge, UK) was added to the culture medium to increase single‐cell survival. Passages for pre‐expansion were performed at 70% confluence using 0.48 mmol/L EDTA (Versene, Gibco^®^ by Thermo Fisher Scientific, Waltham, MA, USA).

### 2.3 Expansion of hiPSCs in 3D bioreactors

Following pre‐expansion, either 10 × 10^6 ^(AS 10) or 50 × 10^6^ (AS 50, LS 50) hiPSCs were inoculated as single‐cell suspension into pre‐coated bioreactors (8.68 µg/cm^2 ^Matrigel, Corning) and cultured over 15 days. The initial cell numbers used in this study are based on previous studies on the hepatic differentiation of hiPSCs in the AS bioreactor, where 100 × 10^6^ cells were inoculated.^15^ Thus, an initial cell number of 10 × 10^6^ cells, resp. 3.3 × 10^6^ cells/mL in AS 10 provides the spatial conditions for at least a 10‐fold cell expansion, while an initial cell number of 50 × 10^6^ resp. a cell density of 16.6 × 10^6^ cells/mL in AS 50 should enable at least a 2‐fold expansion. The latter was chosen to investigate the influence of a high initial cell density on the expansion procedure. For the feasibility testing of an up‐scale of the hiPSC expansion, the LS bioreactor was inoculated with a cell number of 50 × 10^6^ resp. a cell density of 2.9 × 10^6^ cells/mL, as this equals the conditions of AS 10. To ensure single‐cell survival at the beginning of the experiment, 1 mmol/L ROCK inhibitor (Y‐27632; Abcam) was included into the culture medium as bolus injection and was rinsed out within the first 24 hours of bioreactor cultures.

The bioreactors were placed into a heating chamber constantly kept at 37°C. The medium recirculation rate was set to 10 mL/min (AS) resp. 20 mL/min (LS), whereas the medium feed was initially set to 1 mL/h (AS) resp. 2 mL/h (LS) and adapted daily to up to 12 mL/h (AS) or 40 mL/h (LS), depending on the glucose consumption rates. Thereby, glucose levels were kept above 4.4 mmol/L throughout the culture period. The gas perfusion rate was constantly maintained at 20 mL/min (AS) resp. 40 mL/min (LS); CO_2_ was added at a percentage of up to 5% for pH regulation to approximately 7.2 (Table 1).

### 2.4 Biochemical parameters

The metabolic activity of cultured cells was analysed by daily measurements of biochemical parameters in samples from the recirculating medium. Glucose and lactate concentrations were determined by means of a blood gas analyser (ABL 700; Radiometer, Copenhagen, Denmark). Potential cell damage was assessed by measuring the release of lactate dehydrogenase (LDH) using an automated clinical chemistry analyser (Cobas^®^ 8000; Roche Diagnostics, Mannheim, Germany) provided by Labor Berlin GmbH. In addition, beginning differentiation was detected by measurement of alpha‐fetoprotein (AFP) levels in the culture perfusate, using a clinical chemistry analyser (Cobas^®^ 8000; Roche Diagnostics) provided by Labor Berlin GmbH. AFP levels were analysed every 5 days, or more frequently when concentrations were above the detection limit.

### 2.5 CellTiter‐Blue^®^ Cell Viability Assay

Cell activity was evaluated, and cells quantified by performing the CellTiter‐Blue^®^ Cell Viability Assay (CTB; Promega GmbH, Mannheim, Germany) on day 15 of bioreactor cultures and parallel 2D cell cultures. The CTB reagent was applied at a concentration of 2.5% to the bioreactor recirculation with an incubation period of 60 minutes. The feed supply of bioreactors was paused during the measurement period. Samples of 300 µL were taken every 15 minutes from the supernatant and transferred to three wells of a 96‐well plate (Greiner Bio‐One GmbH, Frickenhausen, Germany); further conversion of resazurin to resorufin in the samples was stopped by adding 50 µL of 3% sodium dodecyl sulphate (SDS, Carl Roth GmbH + Co. KG, Karlsruhe, Germany) in dimethyl sulfoxide (DMSO, Sigma‐Aldrich/Merck, Darmstadt, Germany) per well as stop solution. Fluorescence measurements took place at a wavelength of 560 nm (excitation) and 590 nm (emission) using the Infinite M200 Pro plate reader (Tecan Group Ltd., Männedorf, Switzerland). For cell quantification and growth characterization, a calibration curve was generated by correlating defined cell numbers to the resulting CTB gradients (data not shown).

### 2.6 Embryoid body formation

Embryoid bodies were generated by transferring a number of 2 × 10^6^ hiPSCs in 2 mL mTeSR supplemented with ROCK inhibitor (Y‐27632, 1 mmol/L; Abcam) into a well of an AggreWell 800 plate (Stemcell Technologies). The plate was centrifuged at 500 *g* for 3 minutes and incubated overnight at 37°C and 5% CO_2_. On the following day, the formed embryoid bodies were removed from the plate using a trimmed pipette tip with a 1 mL pipette and transferred to wells of non‐treated 12‐well culture plates (Costar^®^, Corning^®^, NY, USA) for expression analysis or to Lumox plates (Sarstedt, Nümbrecht, Germany) for immunohistochemical staining. Also, the mTeSR medium was replaced with E6‐medium,^16^ consisting of 96.8% DMEM‐F12 (Gibco^®^; Thermo Fisher Scientific), 2% insulin‐transferrin‐selenium (Gibco^®^; Thermo Fisher Scientific), 1% Pen Strep (Gibco^®^; Thermo Fisher Scientific) and 0.2% l‐Ascorbic Acid (Sigma‐Aldrich/Merck). Embryoid bodies were cultured over 15 days in total; during the culture period, half of the medium was removed and replaced with fresh E6‐medium three times per week.

### 2.7 Gene expression analysis

Gene expression analysis was performed as described previously^15^, ^17^ using human‐specific primers and probes as listed in Table 2. Expression values of measured genes were normalized to expression values of the housekeeping gene glyceraldehyde‐3‐phosphate dehydrogenase (GAPDH), and fold changes of expression levels were calculated using the ΔΔ*C*_t_ method.^18^

**Table 2:** Applied biosystems TaqMan gene expression assays^®^

Gene symbol	Gene name	Assay ID
*AFP*	Alpha‐fetoprotein	Hs00173490_m1
*CXCR4*	C‐X‐C Motif Chemokine Receptor 4	Hs00607978_s1
*GATA2*	GATA Binding Protein 2	Hs00231119_m1
*NANOG*	Nanog Homeobox	Hs02387400_g1
*NEFL*	Neurofilament Light	Hs00196245_m1
*PAX6*	Paired Box 6	Hs00240871_m1
*POU5F1*	POU Class 5 Homeobox 1	Hs00999632_g1
*SOX17*	SRY‐Box 17	Hs00751752_s1
*T*	T‐Box Transcription Factor T	Hs00610080_m1

### 2.8 Immunhistochemistry analysis

Upon termination of bioreactor cultures, sections of the capillary bed containing cell material were removed and prepared for immunofluorescence staining as described previously.^19^ Nuclei were counterstained with Dapi (blue). The antibodies used for immunohistochemistry are displayed in Table 3.

**Table 3:** Antibodies used for immunofluorescence staining

	Protein symbol	Species	Manufacturer	Final conc. (μg/mL)
Primary antibody				
Alpha‐fetoprotein	AFP	Mouse	Santa Cruz	2
Marker of proliferation	MKI67	Mouse	BD Biosciences	10
Nestin	NES	Rabbit	Santa Cruz	2
POU Class 5 Homeobox 1	POU5F1	Rabbit	Santa Cruz	2
Vimentin	VIM	Rabbit	Santa Cruz	2
Αlpha smooth muscle actin	α‐SMA	Mouse	Sigma‐Aldrich	10‐30
Secondary antibody				
Alexa Fluor 488 anti‐mouse		Goat	Life Technologies	2
Alexa Fluor 594 anti‐rabbit		Goat	Life Technologies	2

### 2.9 Statistical evaluation

Statistical analysis was performed using GraphPad Prism 7.0 for Windows (GraphPad Software, SanDiego, CA, USA). Data are presented as means ± standard error of the mean (SEM) from three or four runs at each inoculum density for AS, or as single values for LS. For evaluation of differences in growth behaviour between AS 10 and AS 50, the areas under curves (AUCs) and the tipping points (ie time when peak values of the curves were reached) were calculated and compared using the unpaired, two‐tailed Student's *t* test. Gene expression data were compared between AS 10 and AS 50, corresponding 2D cultures and embryoid bodies by one‐way analysis of variance (ANOVA). Slope values obtained in the CellTiter‐Blue^®^ Cell Viability Assay as well as cell quantification data, population doublings and doubling times were compared using the unpaired, two‐tailed Student's *t* test.

## 3 RESULTS

### 3.1 Metabolic activity of hiPSCs during bioreactor expansion

**Figure 2:**
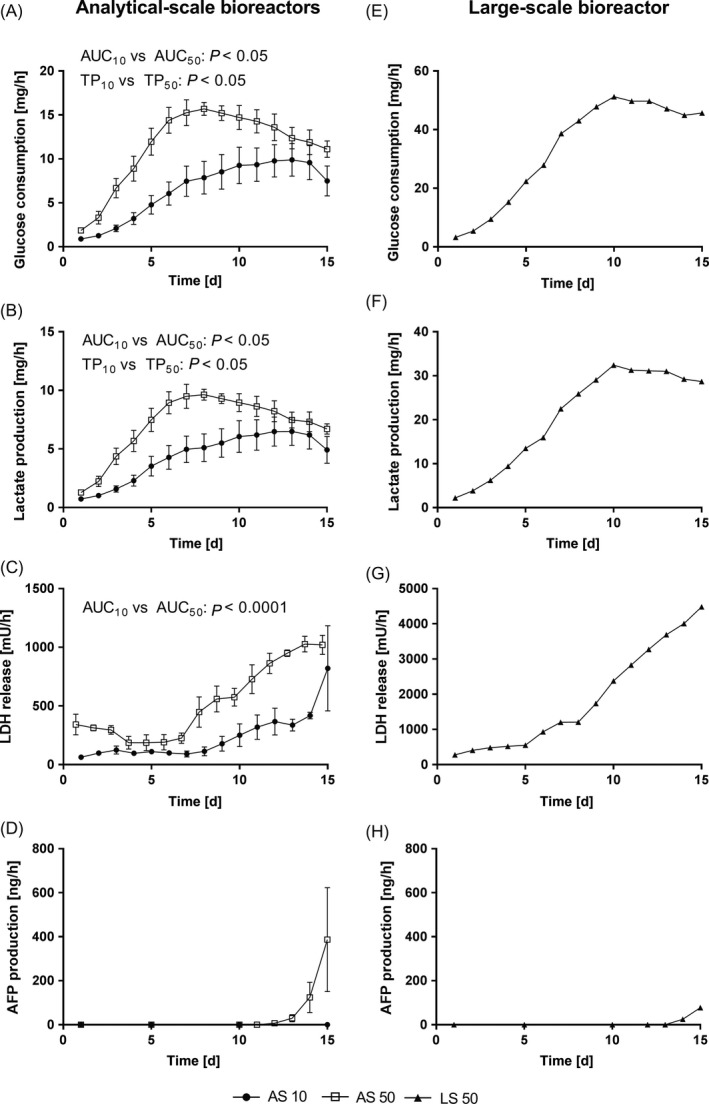
Comparison of clinical chemistry parameters during the culture of human‐induced pluripotent stem cells over 15 days in analytical‐scale bioreactors inoculated with either 10 × 10^6^ (AS 10, *n* = 4) or 50 × 10^6^ (AS 50, *n* = 3) cells, or the large‐scale bioreactor inoculated with 50 × 10^6^ (LS 50, *n* = 1) cells. Values are presented as mean ± SEM (AS 10 and AS 50) or single values (LS 50). The two analytical‐scale bioreactors were compared by means of the areas under the curves (AUCs) and the tipping points (TP). Differences were considered significant at *P* < 0.05

For comparative evaluation of the hiPSC growth behaviour in the two analytical‐scale bioreactors (AS) and the large‐scale bioreactor (LS), glucose and lactate were measured as indicators for the energy metabolism of the cells. Time courses of glucose consumption and lactate production revealed significant differences between AS 10 and AS 50 (Figure 2A,B). The area under curve (AUC) of AS 50 was significantly larger compared with the AUC of AS 10 (*P* < 0.05). Also, the tipping point was achieved significantly earlier in AS 50 with day 7 for both, glucose and lactate, compared with day 12 for glucose and day 11 for lactate in AS 10 (*P* < 0.05 for glucose and lactate). The metabolic parameters for LS 50 (Figure 2E,F) revealed maximum values that were more than three times as high compared with maximum values obtained in AS 50. Release rates of LDH, indicating potential cell death, increased in AS 10 and AS 50 with culture progression, but were significantly higher in AS 50 throughout the culture period compared with AS 10 (Figure 2C; *P* < 0.0001). For LS 50 (Figure 2G), LDH release showed a similar time course as AS 50, while absolute values were three times as high as in AS 50. The albumin precursor AFP, indicating beginning differentiation, showed an exponential increase from day 12 onwards for AS 50 (Figure 2D). In contrast, there was no AFP detectable in perfusates of AS 10 during the entire culture period (Figure 2D). For LS 50 (Figure 2H), a slight increase was measured from day 14 onwards, but maximum values were almost five times lower than those observed in AS 50.

In conclusion, higher cell densities led to a significantly higher overall cell activity in AS bioreactors; however, higher cell densities also led to beginning differentiation as indicated by increasing AFP levels in AS 50. The highest values for energy metabolism were achieved in LS 50, being more than three times as high compared with maximum values obtained in AS 50.

### 3.2 Gene expression profiles of hiPSC cultures

**Figure 3:**
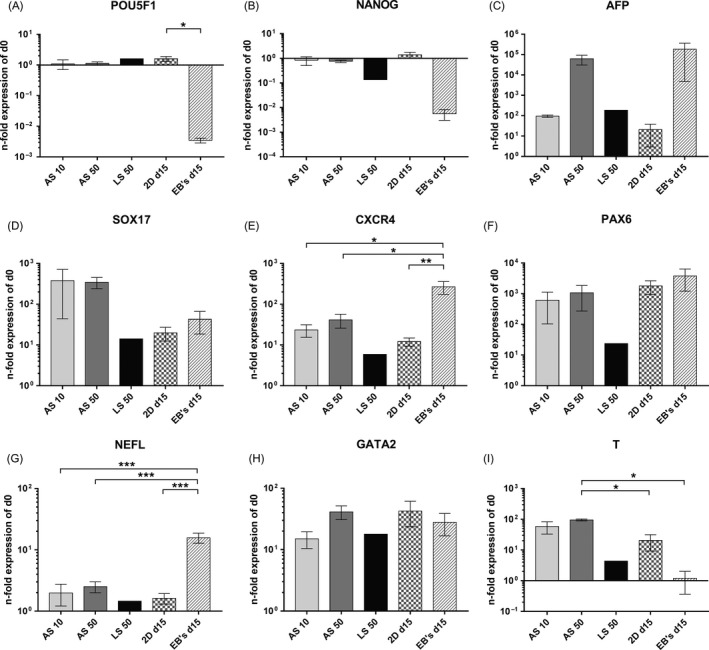
Gene expression analysis after 15 days of hiPSC culture in analytical‐scale bioreactors inoculated with 10 × 10^6^ (AS 10) or 50 × 10^6^ (AS 50) cells, in the large‐scale bioreactor inoculated with 50 × 10^6^ cells (LS 50), on 2D culture plates, or after formation of embryoid bodies. The figure displays gene expression data of POU Class 5 Homeobox 1 (*POU5F1*, [A]), Nanog Homeobox (*NANOG*, [B]), Alpha‐Fetoprotein (*AFP*, [C]), SRY‐Box 17 (*SOX17*, [D]), C‐X‐C Motif Chemokine Receptor 4 (*CXCR4*, [E]), Paired Box 6 (*PAX6*, [F]), Neurofilament Light (*NEFL*, [G]), GATA Binding Protein 2 (*GATA2*, [H]) and T‐Box Transcription Factor T (*T*, [I]). Expression data were normalized to the housekeeping gene Glyceraldehyde‐3‐Phosphate Dehydrogenase (*GAPDH*), and n‐fold expression values were calculated relative to undifferentiated hiPSCs before inoculation on d0 using the ΔΔ*C*_t_ method. Data are presented as mean ± SEM (AS 10 *n* = 3; AS 50 *n* = 3; LS 50 *n* = 1; 2D d15 *n* = 5; EB's d15 *n* = 3). Differences between AS 10, AS 50 as well as 2D cultures and embryoid bodies were detected using the one‐way ANOVA; calculated values were considered significant at *P < 0.05, **P < 0.01 and ***P < 0.001

For the characterization of hiPSCs after expansion in 3D bioreactors, the gene expression of pluripotency as well as differentiation markers relative to the undifferentiated state were analysed. The expression data of the two pluripotency markers *POU5F1* and *NANOG* (Figure 3A,B) revealed only slight changes in pluripotency of bioreactor cultures and 2D cultures compared with the undifferentiated state. For the embryoid bodies, however, a distinct reduction in *POU5F1* and *NANOG* expression was detected, which was significant for *POU5F1* compared with 2D cultures (*P* < 0.05). Regarding differentiation markers, the strongest increases in gene expression were observed for the endodermal lineage marker *AFP* (Figure 3C) with highest values being detected for embryoid bodies and for AS 50. Gene expression measurements for the other two endodermal markers, *SOX17* (Figure 3D) and *CXCR4* (Figure 3E) revealed an increase compared with the undifferentiated state in AS 10 and AS 50. For *SOX17*, the increase was most pronounced in AS 10 and AS 50 and lowest in LS 50. The expression of *CXCR4* showed the highest value for the embryoid bodies, which was significantly higher compared with AS 10 and AS 50 (*P* < 0.05) as well as the 2D cultures (*P* < 0.01). Expression data for the ectodermal marker *PAX6* (Figure 2F) revealed a comparable increase in AS 10 and AS 50, while LS 50 had a noticeable lower increase in *PAX6* expression. The expression data for the second marker of the ectodermal lineage, *NEFL* (Figure 3G), showed the strongest increase for embryoid bodies, with expression values being significantly higher compared with AS 10 and AS 50 as well as the 2D cultures (*P* < 0.001). Expression data for the mesodermal lineage marker *GATA2* (Figure 3H) showed a similar gene expression for all tested groups. In contrast, values for *T* (Figure 3I), another mesodermal marker, revealed the highest expression values in AS 10 and AS 50 and the lowest ones in the embryoid bodies. Expression values of AS 50 were significantly higher compared with 2D cultures and embryoid bodies (*P* < 0.05).

To summarize, gene expression profiles indicate beginning differentiation processes in bioreactor cultures, which were most pronounced in AS 50. However, in embryoid bodies, the expression of both pluripotency markers was lower, and expression of the majority of differentiation markers was higher compared with the bioreactor cultures.

### 3.3 Cell activity of hiPSC cultures

**Figure 4:**
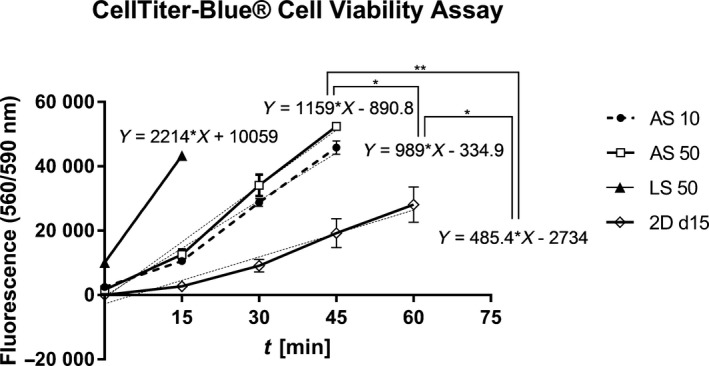
Comparison of CellTiter‐Blue^®^ fluorescence values over a time period of up to 60 minutes on the final day of the experiment (day 15). The figure shows measurements performed in analytical‐scale bioreactors (AS) inoculated with 10 × 10^6^ (AS 10, *n* = 2) or 50 × 10^6^ (AS 50, *n* = 3) cells, the large‐scale bioreactor (LS) inoculated with 50 × 10^6^ cells (LS 50, *n* = 1) and 2D cultures (2D d15, *n* = 4). Differences in the gradients of the corresponding linear correlation were detected using the unpaired, two‐tailed Student's *t* test and considered statistically significant at *P < 0.05 and **P < 0.01

The CellTiter‐Blue^®^ Cell Viability assay (CTB) was performed in all bioreactors as well as in 2D cultures, which were cultured in parallel to bioreactor cultures (Figure 4). The strongest increase during the assay performance was detected in LS 50. The curves of AS 10 and AS 50 were comparable, with the slope of AS 50 being significantly larger than the slope of AS 10 (*P* < 0.05). The curve of 2D cultures showed the lowest fluorescence values with a significantly smaller slope compared with AS 10 (*P* < 0.05) and AS 50 (*P* < 0.01). Fluorescence measurements were above the detection limit after 15 minutes (LS 50) or 45 minutes (AS 10 and AS 50) and are therefore not included in the graph.

In conclusion, the highest cell activity was detectable in LS 50, followed by AS 50 and AS 10. The increase in cell activity of AS 50 was significantly larger than that of AS 10.

### 3.4 Immunohistochemical characteristics of hiPSC cultures

**Figure 5:**
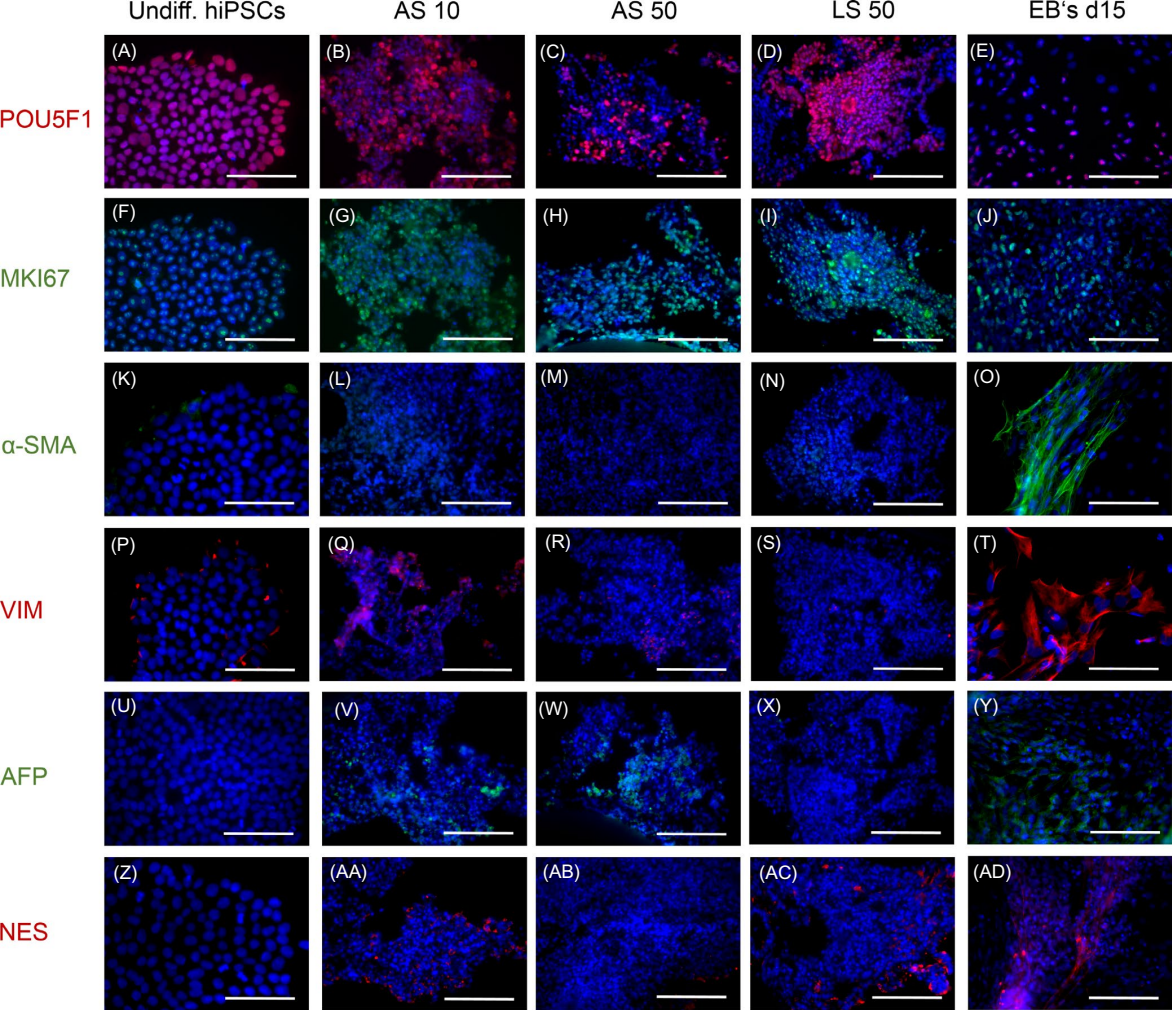
Immunohistochemical staining of undifferentiated human‐induced pluripotent stem cells (hiPSCs) and hiPSCs after culture in analytical‐scale bioreactors (AS) inoculated with 10 × 10^6^ cells (AS 10) or 50 × 10^6^ cells (AS 50), in the large‐scale bioreactor (LS) inoculated with 50 × 10^6^ cells (LS 50) and in embryoid bodies (EB's d 15). The figure shows staining of POU Class 5 Homeobox 1 (POU5F1, A‐E), marker of proliferation (MKI67, F‐J), α‐smooth muscle actin (α‐SMA, K‐O) and vimentin (VIM, P‐T), alpha‐fetoprotein (AFP, U‐Y) and nestin (NES, Z‐AD). Nuclei were counterstained with Dapi (blue). Scale bars correspond to 100 µm

Staining with the pluripotency marker POU5F1 (Figure 5A‐E) and the proliferation marker MKI67 (Figure 5F‐J) showed that undifferentiated cells and the vast majority of cells in AS 10 or LS 50 were positive for POUF51 and MKI67. In contrast, only approximately half of the cells in AS 50 were positive for those markers. Staining of the embryoid bodies revealed about one third of the cells to be positive for POU5F1 and MKI67. The markers for the mesodermal lineage, α‐SMA (Figure 5K‐O) and vimentin (Figure 5P‐T), were clearly positive in embryoid bodies and interestingly, the stained structures appeared filament‐like. Staining of α‐SMA in all other groups was mostly negative, whereas vimentin was positive in a few cells in undifferentiated hiPSCs and bioreactor cultures. The marker for the endodermal lineage AFP (Figure 5U‐Y) was detectable in the majority of cells in the embryoid bodies and in parts of cells in AS 10 and AS 50. In contrast, undifferentiated hiPSCs and cells in LS 50 appeared negative for AFP. The marker for the ectodermal lineage, nestin (Figure 5 Z‐AD) was detected in embryoid bodies and, again, filament‐like structures were visible. A small amount of cells in AS 10 or LS 50 were positive, whereas in AS 50, almost all cells were negative for nestin. In undifferentiated hiPSCs, nestin was not detectable.

In summary, the majority of cultured cells in bioreactors expressed both, the pluripotency marker and the proliferation marker, whereas only a small fraction of cells was positive for differentiation markers. Cells cultured as embryoid bodies showed a more distinct expression of differentiation markers, partially forming filament‐like structures.

### 3.5 Proliferation yields of hiPSCs expanded in bioreactors or 2D cultures

Based on the results of the CTB and a corresponding calibration curve obtained from 2D cultures (data not shown), cell numbers were determined on the final day of the bioreactor experiments or the final day of 2D cultures (Table 4). For AS 10, a mean cell number of 1.01 × 10^9^ ± 21.04 was achieved on day 15, which was significantly lower compared with the cell yield in AS 50 with cell numbers of 1.40 × 10^9^ ± 37.96 (*P* < 0.05). Cells cultured in LS 50 were expanded to 5.4 × 10^9^ cells, which is the highest achieved cell number. The determined cell numbers at the end of the 15‐day bioreactor culture revealed an over 100‐fold increase in cell number for AS 10 and for LS 50. In contrast, AS 50 showed a 28‐fold increase. The achieved cell densities were 3.66 × 10^8^ cells/mL for AS 10, 4.69 × 10^8^ cells/mL for AS 50 and 3.17 × 10^8^ cells/mL for LS 50. The population doubling of bioreactor cultures was similar in AS 10 (6.77 ± 0.03) and LS 50 (6.74), resulting in doubling times of 2.22 ± 0.01 days resp. 2.23 days. AS 50 revealed a significantly lower overall population doubling with 4.80 ± 0.04 (*P* < 0.001) and a significantly longer doubling time with 3.12 ± 0.02 days (*P* < 0.001) compared with AS 10. In 2D cultures, a cell number of 49.49 × 10^6^ ± 9.02 was achieved after a 15‐day culture period, with a population doubling of 7.16 ± 0.30 and an average doubling time of 2.10 ± 0.09 days.

**Table 4:** Cell quantification and growth characterization of cells cultured in bioreactors or 2D cultures after 15 days

	AS 10	AS 50	LS 50	2D
Cell number (mio)	1097.73 ± 21.04*	1404.58 ± 37.96*	5394.25	49.49 ± 9.02
Population doubling	6.77 ± 0.03***	4.8 ± 0.04***	6.74	7.16 ± 0.30
Doubling time (days)	2.22 ± 0.01***	3.12 ± 0.02***	2.23	2.10 ± 0.09

Differences between AS 10 and AS 50 bioreactors were considered significant at

* *P*<0.05 and

*** *P*<0.001.

To conclude, a more than 100‐fold increase in cell number was achieved in AS 10 and LS 50, whereas a 28‐fold increase was reached in AS 50. Population doublings and doubling times reflected these results.

## 4 DISCUSSION

Since the application of hiPSCs in the medical field requires large cell quantities at high‐quality standards, it is of great interest to evaluate factors that influence hiPSC expansion in 3D culture systems. Therefore, the effect of the inoculum density on the hiPSC expansion procedure, cell differentiation and the cell yield was investigated in this study.

For evaluation of the energy metabolism of hiPSCs cultured in bioreactors, glucose consumption and lactate production were determined. Analytical‐scale (3 mL) bioreactors inoculated with 50 × 10^6^ cells (AS 50) consumed glucose faster than analytical‐scale bioreactors inoculated with 10 × 10^6^ cells (AS 10), and growth stagnated significantly earlier. This observation can primarily be explained by the higher initial cell density in AS 50, resulting in higher overall glucose uptake and metabolic activity, but also by an increased cell‐cell signalling, both leading to an increased cell proliferation and expansion rate.^20^, ^21^ Similar findings have been reported by Meng et al,^22^ who observed the strongest increase in viable cell density at the highest cell inoculation number when inoculating three different cell densities in shape of cell aggregates into stirred suspension bioreactors. Additionally, Abaci et al^23^ observed a sharp decrease in oxygen concentration in human embryonic stem cell (hESC) and iPSC cultures as a result of high cell seeding densities, indicating a corresponding increase in energy metabolism.

The observed shift from cell expansion towards maintenance, which occurred the earliest in AS 50 as indicated by the tipping point, is in line with results reported by Simmons et al,^24^ who detected a plateau phase in cell growth and glucose consumption rates after approximately 1 week during 3D culture of rat mesenchymal stem cells. The observed stagnation of cell growth in that study was ascribed to the limited space for the cells in the 3D fibre mesh scaffolds which were placed into a flow perfusion bioreactor. Since AS 50 has the highest initial cell density in relation to the size of the bioreactor cell compartment, a growth stagnation due to space limitations appears likely in our study, too. However, growth limitations could also be caused by depletion of nutrients and oxygen in cell aggregates of larger size,^25^ leading to cell differentiation or cell damage.^26^ In order to evaluate potential cell damage and cell differentiation during cell culture, levels of LDH, an enzyme released during loss of plasma membrane integrity of cells,^27^, ^28^ and alpha‐fetoprotein (AFP), a marker for endodermal differentiation,^29^ were measured in the perfusates of bioreactor cultures. In AS 50 (*n* = 3), release of LDH was significantly higher compared with the LDH release in AS 10 (*n* = 4), which could be a result of both, upper cellularity limit of the cell compartment and/or large aggregate size. The release of AFP in AS 50 and LS 50 towards the end of the culture indicates beginning differentiation of the cells,^30^ in parallel with the long plateau phase of approximately 8 resp. 5 days. Beginning differentiation was also indicated by the mRNA expression analysis, which was performed upon termination of bioreactor cultures. An upregulation of especially endodermal differentiation markers has been previously reported for 3D cultures of mouse embryonic stem cells by Knöspel, Freyer et al and was ascribed to reduced oxygen and nutrient supply in the centre of cell aggregates, amongst others.^17^ A large aggregate size may further explain elevated expression levels of *SOX17*, *CXCR4*, *PAX6*, *NEFL*, *GATA2* and *T* indicating a beginning undirected differentiation of hiPSCs. The tendency of elevated gene expression of differentiation markers, which occurred especially in AS 50, is in line with findings reported by Toyoda et al,^31^ who observed that the differentiation of hiPSCs into pancreatic bud‐like progenitor cells was enhanced by high cell densities. However, for *CXCR4* and *NEFL*, expression levels of embryoid bodies were significantly higher than expression levels of the bioreactor or 2D cultures, indicating that the differentiation processes are minor compared with intended differentiation as performed in embryoid body cultures. Interestingly, the expression analysis for *T* revealed significantly lower expression levels for embryoid bodies compared with AS 50 and 2D cultures. Maximum levels for *T* in embryoid bodies built of human embryonic stem cells were measured between day 3 and 7,^32^, ^33^, ^34^ which explains the low levels of *T* expression in embryoid bodies in this study, which were analysed on day 15. Also, as *T* is a marker for early mesodermal differentiation, the hypothesis is supported that bioreactor cultures only show a beginning differentiation. Furthermore, immunohistochemical staining did not show a strong expression of any of the differentiation markers, especially when being compared with the staining patterns of the embryoid bodies. Nevertheless, beginning differentiation as well as cell death induced by cell compartment size limitations or aggregate size during hiPSC expansion could potentially be avoided by either harvesting cells from the cell compartment,^17^ or by performing passages during continuous expansion in 3D culture systems^26^ as soon as cell cultures reach a growth plateau.

Results from the CellTiter‐Blue^®^ Cell Viability Assay underlined the results from glucose and lactate measurements with the additional finding that all bioreactors showed higher cell activity compared with corresponding 2D cultures, emphasizing the use of 3D culture systems instead of 2D cultures for hiPSC long‐term expansion.

Studies on hiPSC expansion have been published using different culture models and inoculum densities, emphasizing that the ideal inoculum density and expansion efficiency varies depending on specific culture characteristics. For example, Kropp et al^35^ used an inoculum density of 5 × 10^5^ hiPS cells/mL for expansion in stirred tank bioreactors, whereas Olmer et al^36^ used an initial cell density of only 5 × 10^4^ hiPS cells/mL for expansion in stirred bioreactors. In these studies, hiPSC proliferation by the 4‐ to 6‐fold was observed, reaching 2 × 10^6^ to 3.5 × 10^6^ cells/mL.^35^, ^36^ In contrast, Lei et al^37^ applied a hydrogel‐based 3D culture system with inoculum densities between 2.5 × 10^5^ and 2.5 × 10^6^ cells/mL and reported a 20‐fold hiPSC expansion with a resulting cell density of 2 × 10^7 ^cells/mL. The results of the present study show that the used four‐compartment bioreactor enables a 100‐fold expansion, reaching 4.69 × 10^8^ cells/mL in the bioreactor compartment. Initially developed for use as an extracorporeal liver support system and successfully applied as such,^38^ the compartmentalized bioreactor provides a countercurrent “arteriovenous” media flow and decentralized gas perfusion via capillaries, thereby enhancing mass exchange for an optimized nutrient and oxygen supply for the cultured cells.^39^ Also, cells are not affected by shear stress, which occurs for example if cells are cultured in stirred tank bioreactors,^40^ and can cause cell damage.^41^

The total amount of cells produced in LS 50 (5.4 × 10^9^ cells) would suffice for single‐patient treatments in heart and liver therapies as well as treatment of diabetes.^11^ Cell numbers of this relevance have to date been only achieved by Kwok et al,^42^ who produced 2 × 10^9^ hiPSCs after 14 days of stirred suspension culture, and Abecasis et al,^26^ who obtained 10^10^ pluripotent hiPSCs within 11 days of 3D culture and three sequential passages. The implementation of a dissociation protocol into our studies may even further improve the expansion rates and the maintenance of an undifferentiated state of cultured hiPSCs for long‐term expansion procedures. Also, a larger number of repetitions, especially of the LS 50 run, are needed in order to verify the results of the study.

Several research groups observed that higher cell densities support differentiation processes of pluripotent stem cells.^31^, ^36^, ^43^, ^44^, ^45^, ^46^, ^47^ However, the majority of studies were performed in 2D culture models, where medium is usually exchanged discontinuously, and cells are limited to growing horizontally, instead of three‐dimensionally. In contrast, perfused 3D cultures enable a continuous supply with nutrients and oxygen, while maintaining cell pluripotency.^48^ In particular, the four‐compartment hollow‐fibre bioreactors used in this study aim to mimic the in vivo situation in the tissue, thereby enabling increased cell densities at physiological levels.^17^ Therefore, the results gained in 2D cultures may not be directly comparable to the 3D cultures used in the present study.

Furthermore, the results presented in 2D studies regarding critical cell densities varied, depending on the initial cell type and the desired differentiation outcome. For example, Selekman et al^49^ found that a human pluripotent stem cell density of 6500 cells/cm^2^ is optimal for an epithelial differentiation considering the balance between purity and yield of cells. In contrast, initial seeding densities of dental and oral stem cells for neural induction in 2D cultures laid between 3000 cells/cm^2^ and 20 000 cells/cm^2^.^50^

Overall, the expansion of hiPSCs in 3D hollow‐fibre bioreactors was successful for different cell inoculation conditions and bioreactor sizes. The use of a larger bioreactor (17 mL) resulted in clinically relevant cell yields. The findings also show that the inoculum density has significant influence on the growth behaviour and the differentiation state of the cells in 3D bioreactors. A high cell inoculation number led to a faster expansion with higher maximum values for the glucose uptake and growth, but also to cells more prone to differentiation. In contrast, lower initial cell numbers led to slower expansion, but showed less differentiation and required less time and effort for pre‐expansion of the inoculum to the 3D bioreactor. The latter is especially of relevance for efficient hiPSC expansion in large‐scale bioreactors. Based on the described results, we conclude that 3D perfusion bioreactors should be inoculated with low cell numbers for achieving a successful long‐term hiPSC expansion for clinical purposes. In order to avoid differentiation, additional repeated cell harvesting or cell aggregate dissociation may be included into the expansion procedure.

## CONFLICT OF INTEREST

The authors have no conflict of interest to declare.

## AUTHOR CONTRIBUTIONS

SG, NF, KZ, TM and CFM designed the experiments. SG, NF, GH and MB performed the experiments. SG and NF analysed the data. SG wrote the manuscript. FS was responsible for technical developments and bioreactor prototype design. MS supervised the study procedure. NF, KZ, MS, TM and CFM critically revised the manuscript.
